# Safety and performance of the HYBRIDknife flex in a porcine model of esophageal endoscopic submucosal dissection: A pilot study

**DOI:** 10.1002/jgh3.70036

**Published:** 2024-11-29

**Authors:** Christopher J L Khor, Katsuro Ichimasa, Stephen K K Tsao, Ulrich Biber, Yutaka Saito

**Affiliations:** ^1^ Singapore General Hospital Singapore and Duke‐NUS Medical School Singapore Singapore; ^2^ Showa University Northern Yokohama Hospital Yokohama Japan; ^3^ Aliveo Medical Singapore Singapore; ^4^ Erbe Elektromedizin GmbH Tuebingen Germany; ^5^ Endoscopy Division National Cancer Center Hospital Tokyo Japan

**Keywords:** electrosurgery, endoscopic resection, endoscopic submucosal dissection, gastrointestinal endoscopy, glycerol

## Abstract

**Background and Aim:**

Endoscopic submucosal dissection (ESD) is considered the best modality for achieving en bloc resection of larger neoplastic mucosal lesions in the upper and lower gastrointestinal (GI) tract. Multiple devices are available for ESD, and refinements continue to be made to develop devices that improve the safety and efficiency of performing ESD. Submucosal injection with viscous fluids like glycerol, which prolong submucosal expansion, could facilitate the procedure. We aimed to evaluate the safety and performance of the new Erbe HYBRIDknife® flex, which combines electrosurgical dissection with waterjet‐assisted injection in a slim and flexible form factor.

**Methods:**

In a prospective animal study with six pigs, four endoscopists, each with 10–20 years of experience in ESD, performed 28 esophageal ESDs. One half was performed with physiological saline injectate, the other half with fructose‐added glycerol. Various performance aspects were evaluated on a five‐point scale [5 = best], including dissection properties, handling, and usability.

**Results:**

No perforations or major bleeding occurred. All resections were performed en bloc, with one technical failure (3.6%, 1 of 28). Performance scores were similar for saline and glycerol (4.5 ± 0.31 vs. 4.5 ± 0.32, *P* = 0.36), as was dissection speed (13 ± 6.2 mm^2^/min vs. 15 ± 6.1 mm^2^/min, *P* = 0.22).

**Conclusions:**

We demonstrated that esophageal ESD can be performed safely and rapidly using HYBRIDknife flex, with excellent performance evaluation by the endoscopists. Combining this device with glycerol or saline is precise and effective for ESD, although experience could compensate for the theoretical disadvantage of using normal saline.

## Graphical abstract

This study showed that Esophageal ESD could be performed precisely, safely and rapidly with the new Erbe HYBRIDknife® flex in a porcine model. No difference was seen in the performance metrics between the use of saline and glycerol as the injectate.

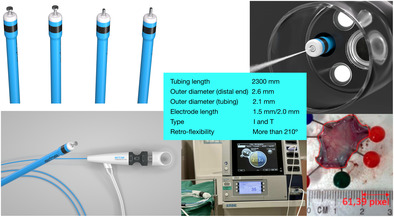

## Introduction

A variety of methods and techniques are available for the endoscopic resection of GI lesions. ESD has emerged as the minimally invasive technique of choice for en bloc resection of early neoplastic GI lesions,^1^, ^2^ especially for lesions >2 cm in diameter, and with wide adoption in East Asia.^3^, ^4^, ^5^, ^6^, ^7^ Compared with piecemeal endoscopic mucosal resection (p‐EMR), ESD enables improved pathological assessment of the depth of carcinoma invasion, lympho‐vascular invasion and determination of lateral and vertical resection margins with reduced local recurrence rate.^6^, ^8^ ESD, however, is more technically demanding than p‐EMR, more difficult to learn, and offers fewer training opportunities.^9^, ^10^ The choice of ESD device may play a role in achieving the determinants of success in ESD, namely en bloc resection and the avoidance of complications such as perforation and bleeding. The HybridKnife® combines a through‐needle high‐pressure waterjet with an electrosurgical instrument and was designed for GI ESD procedures. It has been evaluated in both animal studies^10^, ^11^, ^12^, ^13^ and human clinical trials.^13^, ^14^, ^15^, ^16^ This device supports the entire multi‐step procedure without the need for changing devices, as it is capable of marking the lesion, submucosal injection, circumferential mucosal incision, submucosal dissection, and hemostasis.^17^, ^18^ Its use may therefore reduce procedure time^15^, ^16^, ^18^ and perforation rate.^12^ ESD requires an especially high level of expertise when the target lesion is fibrotic, large, sited where the muscularis propria is thin, or where endoscope stability and optimal positioning are difficult to achieve. A combination of these factors may result in an even greater degree of resection difficulty.^19^, ^20^ In such difficult ESDs, high procedural accuracy is essential to avoid complications like perforation and bleeding.^21^ To protect the muscularis from injury and perforation, submucosal cushioning is essential. The use of a more viscous fluid like fructose‐added glycerol or hyaluronate sodium can help make mucosal elevation greater and long‐lasting than with normal saline.^13^, ^22^ The HybridKnife is unique in having a through‐needle high‐pressure saline injection capability, allowing frequent submucosal injections without interruption of dissection, replicating the effect of intermittent injection of a viscous solution. The HybridKnife, however, is not extensively used for difficult ESDs such as those in the colorectum, as the electrode thickness (0.7 mm) and stiffness of the probe may be obstructive; this may not be adequately compensated for by the advantage of high‐pressure injection.

With this in mind, a new device for ESD, the HYBRIDknife flex T‐Type (HK‐T), has been developed to enhance procedural precision. The HK‐T has a thin 0.5‐mm electrode that facilitates fine cutting, and therefore should be particularly suitable for this purpose. It is also much more flexible, which is important when working retroflexed in the stomach and colorectum. In the esophagus, the precision of cutting with a thin electrode and a slimmer sheath (2.1 mm in diameter vs. 2.3 mm in the HybridKnife), which allows better suction in the standard 2.8‐mm working channel, are features that potentially enhance performance, while knife flexibility is expected to be less important in this location. Jacques et al. compared the utility of HK‐T with the Olympus DualKnife™ J in colorectal ESD in an animal trial.^23^ They found the HK‐T to be as effective as the DualKnife J in dissection quality, but also noted that HK‐T had better injection and hemostatic properties. We believe that there are substantial differences between performing ESD in the colon and in the esophagus, as described above, and therefore conducted a pilot study in an *in vivo* porcine model to evaluate the safety and performance of the HK‐T in esophageal ESD.

## Methods

### Study design and test animals

We aimed primarily to evaluate the safety of the new HK‐T during ESD in a pilot study. Secondary aims were to evaluate efficacy and to compare the utility of glycerol as a submucosal lifting agent with normal saline, applied via the HK‐T. Six female Yorkshire‐Landrace pigs (*Sus scrofa domesticus*) aged 11–15 weeks with a mean body weight of 66 ± 2.6 kg (range 62–70 kg) were used. Twenty‐eight esophageal ESDs were performed by four highly experienced endoscopists (four authors), who had performed ESD for 10–20 years. None of the endoscopists had any experience of using the HK‐T prior to the study. Four to five ESDs were performed in each pig. We used either normal saline (0.9% sodium chloride; Baxter, Deerfield, USA; hereafter “saline”) or a fructose‐added glycerol solution (10% glycerol, 5% fructose and 0.9% sodium chloride; Glycereb®, Terumo, Tokyo, Japan; hereafter “glycerol”) for submucosal injection, with Indigo Carmine (Fujifilm, Tokyo, Japan) added. Fourteen ESDs were assigned to each injectate, and the endoscopists were blinded to its usage. Consequently, each endoscopist performed three to four ESDs per injectate, seven ESDs in total. The order of performance was randomized with regards the injectate. Endoscopists were instructed to perform esophageal en bloc ESDs of a simulated 2‐cm ovoid lesion marked by coagulation with a 20‐mm snare (SnareMaster™ SD‐230 U‐20; Olympus, Tokyo, Japan) and the HK‐T. A short transparent cap (ST Hood Short Type; Fujifilm, Tokyo, Japan) was attached to the gastroscope tip. The gastroscope (GIF‐HQ190; Olympus, Tokyo, Japan) was placed in the desired position, after which the HK‐T was inserted. Instrument insertion, marking of the pseudo‐lesion, mucosal elevation by submucosal injection, circumferential mucosal incision, and submucosal dissection, coagulation of blood vessels during/after resection if necessary, and retrieval of the resection specimen comprised a single esophageal ESD. Most ESD devices require an endoscopic injection needle for initial mucosal puncture before submucosal injection, whereas the HK‐T incorporates a high‐pressure waterjet capable of mucosal puncture. Instruments were therefore only changed at the endoscopist's request.

### Study devices and data collection

**Figure 1:**
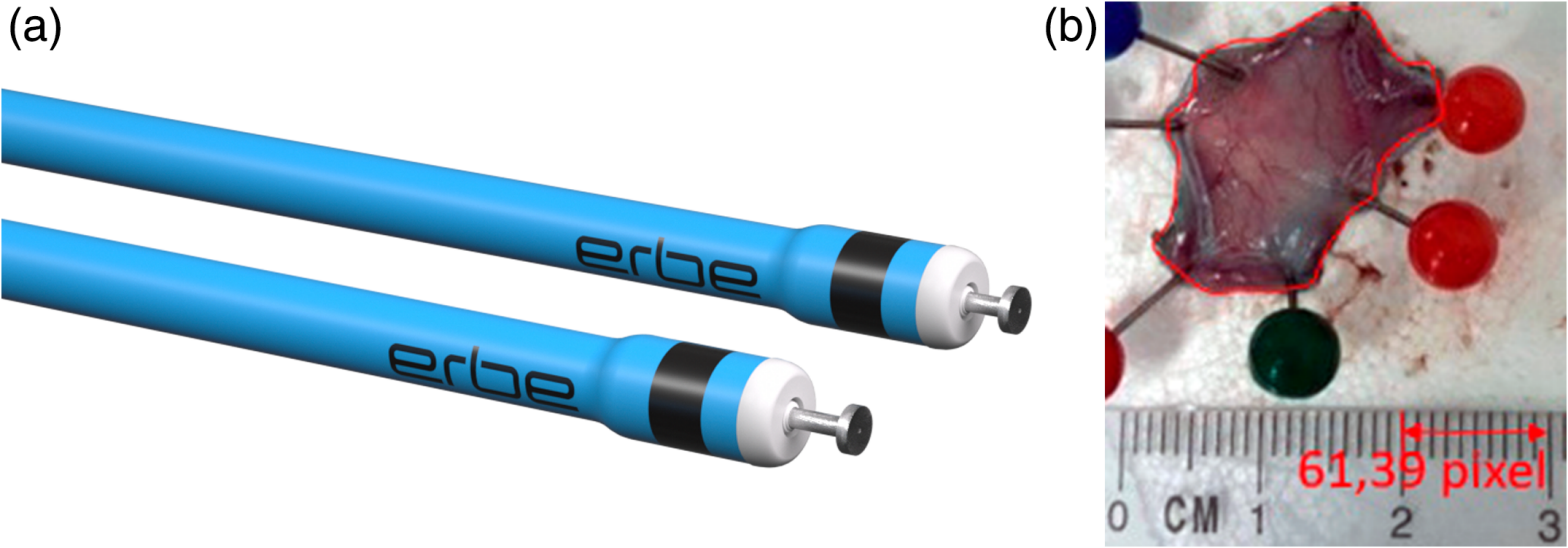
(a) The HYBRIDknife flex T‐Type (Erbe Elektromedizin GmbH, Tuebingen, Germany) with an axial design and an electrode length of 1.5 mm (top) or 2.0 mm (bottom) when projected (2.0 mm was used). An ultrafine waterjet can be delivered through a central capillary at the tip with a pressure of up to 80 bar. The “T” in T‐Type describes the disc‐shaped tip of the electrode. There are also two variants with a needle‐shaped electrode, the I‐Type. (b) Example of an esophageal resection specimen for *ex vivo* evaluation: the diameter was calculated from the surface area described by the circumference of the specimen. Used with permission granted by Erbe Elektromedizin GmbH.

The HK‐T (Fig. 1a) system consists of an electrosurgical knife and an electro−/hydrosurgical supply unit with generator algorithms and settings (Table 1). Detailed dimensions of the HK‐T are stated in Table 1. For waterjet injection with ERBEJET® 2, a pressure setting between Effect 30 and 50 was allowed, starting with Effect 35 as default. For the VIO® 3 electrosurgical unit, variation was allowed to account for endoscopist preferences and esophageal location. Preoperatively, the endoscopists were informed about the interview and the performance aspects (Table 2). After each ESD, device performance was evaluated on a five‐point Likert scale (Table 2). The definitions of the Likert points (LP) were assigned inversely: 1 = Very poor, 2 = Poor, 3 = Neutral, 4 = Good, and 5 = Very good. The average performance score per ESD was the mean of the corresponding 15 individual ratings. The performance scores for saline and glycerol were calculated as the grand average of the 14 mean performance scores per ESD. Procedure time was defined in minutes between the marking of the pseudo‐lesion and the end of dissection (final electrosurgical activation). Complications, that is, minor bleeding, major bleeding requiring the use of a hemostatic forceps, and endoscopically visible perforations were counted per ESD. The injection volume per ESD via the waterjet system and, if used, the syringe combined with the endoscopic injection needle, was measured in milliliters. After each procedure, the success of en bloc resection was checked. Each resection specimen was fixed with pins on Styrofoam board and photographed (Fig. 1b). The specimen diameter was calculated from the dissection area in mm^2^, defined by the circumference of the specimen. For this, a microscope software was used (ZEISS ZEN core v2.7, Carl Zeiss Microscopy GmbH, Jena, Germany). The dissection speed was defined as dissection area divided by procedure time.

**Table 1:** ESD system and settings

ESD knife	Electro‐/Hydrosurgical unit	Modes/Settings
HYBRIDknife flex T‐Type (HK‐T, REF: 20150–111) Working length: 2.3 m Sheath Ø: 2.1 mm Tip specifications [mm]: 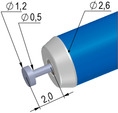	Erbe VIO® 3 (REF 10160–000, software version: 1.3.4) + ERBEJET® 2 (REF 10150–000, software version: 1.1.5)	Marking of pseudo‐lesion: forcedCOAG® Effect 1.0–7.0 (20 W–90 W)
		Incision: endoCUT® I Effect 2 (90 W), cutting duration 3 (100 ms), cutting interval 3 (720 ms)
		Dissection: endoCUT® I like for incision or preciseSECT Effect 5.0–7.0 (64 W–83 W) or dryCUT Effect 4.0–4.7 (81 W–102 W, for stronger hemostasis)
		Injection: ERBEJET® 2: Effect 30–50 & endoscopic injection needle if requested (Olympus REF: NM 400 L‐0421)
		Coagulation: softCOAG® Effect 4.5 (80 W)

ESD, endoscopic submucosal dissection.

**Table 2:** Survey for evaluation of device performance

Performance characteristic	Statement
Insertion of instrument	Insertion of the instrument was completely smooth and without resistance.
Marking of pseudo‐lesion	Coagulation marks were effectively visible.
Injection capability	Injection ability of the instrument was optimal.
Cutting performance	Overall cutting performance was optimal.
Circumferential cutting	The instrument was ideal for circumferential cutting.
Dissection and Thermal spread	Dissection in terms of thermal spread was optimal.
Dissection and Tissue sticking	Dissection in terms of tissue sticking was optimal.
Hemostasis	Hemostasis was optimal and required no additional intervention.
Stability of angulation	The endoscope maintained the chosen angulation with inserted instrument.
Stability during dissection	The instrument could be moved through the target tissue perfectly smoothly during dissection.
Stability of electrode length	The chosen electrode length was stable throughout the procedure.
Suction performance	The suction worked sufficiently with inserted instrument.
Visibility of electrode	The electrode was consistently visible during the procedure.
Visibility of operation field	Visibility of the operation field was not impaired by the instrument.
Working speed	The working speed was optimal.

Response format: 1 = Totally agree, 2 = Agree, 3 = Undecided, 4 = Disagree, 5 = Totally disagree.

### Animal husbandry

The pigs were obtained from the National Large Animal Research Facility, Singapore and identified with ear tags. Premedication was administered by intramuscular injection of atropine (0.05 mg/kg; Atropine Sulphate Atlantic, Vana Corp Ltd., Bangkok, Thailand), azaperone (4.0 mg/kg; Alfaxan®, Jurox, Rutherford, Australia), ketamine (14.0 mg/kg; Ceva Ketamine Injection, Ceva Animal Health, Auckland, New Zealand), and midazolam (1.0 mg/kg; Dormicum®, Cenexi, Fontenay‐sous‐Bois, France). Before endotracheal intubation, 2.0–5.0 mg/kg propofol (Fresenius Kabi, Graz, Austria) was injected intravenously. Intraoperatively, the animals were ventilated and kept under deep anesthesia with isoflurane (0.8–1.6 vol%, Attane™, Piramal Critical Care Inc., Bethlehem, USA) and fentanyl (25–100 μg/kg/h, Durogesic®, Janssen‐Cilag N.V., Beerse, Belgium) for analgesia by intravenous administration. The intraoperative monitoring included electrocardiography, pulse oximetry, and capnometry. Postoperatively, the animals were painlessly euthanized with pentobarbitone sodium (150 mg/kg; Valabarb®, Jurox, Rutherford, Australia).

### Statistical analysis

Prism version 7.05 (GraphPad Software, San Diego, CA 92108, USA) was used for statistics. The performance scores were continuous and normally distributed according to a KS‐Test for each injectate. Therefore, differences were determined using an unpaired one‐sided Student's *t*‐test to identify improvements with glycerol compared with saline. Scores on single performance characteristics (Table 2) are categorical and were compared with a two‐sided Mann–Whitney rank test. The normal distribution of procedure time and dissection speed was likewise evaluated with a KS‐Test. Differences were determined with an unpaired one‐sided Student's *t*‐test for normally distributed data (dissection speed) or with Mann–Whitney‐*U*‐test for not normally distributed data (procedure time) to determine improvements with glycerol. The rationale for using one‐sided tests is based on previous results indicating superiority of glycerol over saline regarding device performance and working speed.^13^ Dichotomous data (incidence of perforation and bleeding) were analyzed with a two‐sided Fisher's exact test. *P*‐values <0.05 were considered statistically significant.

## Results

### Baseline characteristics

The ESD resection specimen diameter ranged from 1.4 to 2.8 cm, and only one resection specimen in the saline group was smaller than the minimum 1.5‐cm diameter specified. There were no significant differences in ESD diameter between the two injection fluids (Table 3; saline: 1.9 ± 0.4 cm vs. glycerol: 2.1 ± 0.4 cm, *P* = 0.25). Similarly, the injection volume was not significantly different between the injection fluids (Table 3, saline: 56 ± 34 mL vs. glycerol: 50 ± 26 mL, *P* = 0.99). The mean injection volume per dissection area was approximately 50% higher using saline (21 ± 16 mL/cm^2^) compared with glycerol (14 ± 6.9 mL/cm^2^), but this difference was not statistically significant (*P* = 0.15).

**Table 3:** Resection size and injection volume from 28 esophageal ESD procedures, 14 ESDs per group

	ESD specimen area (cm^2^)			ESD specimen diameter (cm)			Test statistic
	Total	Saline	Glycerol	Total	Saline	Glycerol	*P*‐Value
Mean (SD)	3.4 (1.3)	3.0 (1.3)	3.7 (1.3)	2.0 (0.4)	1.9 (0.4)	2.1 (0.4)	0.25
Range	1.6–6.0	1.6–6.0	2.1–5.3	1.4–2.8	1.4–2.8	1.6–2.6	

	Injection volume per ESD procedure (mL)			Test statistic	Injection volume per dissection area (mL/cm^2^)			Test statistic
	Total	Saline	Glycerol	*P*‐Value	Total	Saline	Glycerol	*P*‐Value
Mean (SD)	53 (30)	56 (34)	50 (26)	0.99	18 (12)	21 (16)	14 (6.9)	0.15
Range	15–106	15–101	15–106		3.7–55	5.2–55	3.7–23	

ESD, Endoscopic submucosal dissection; SD, standard deviation.

### Key outcomes

**Figure 2:**
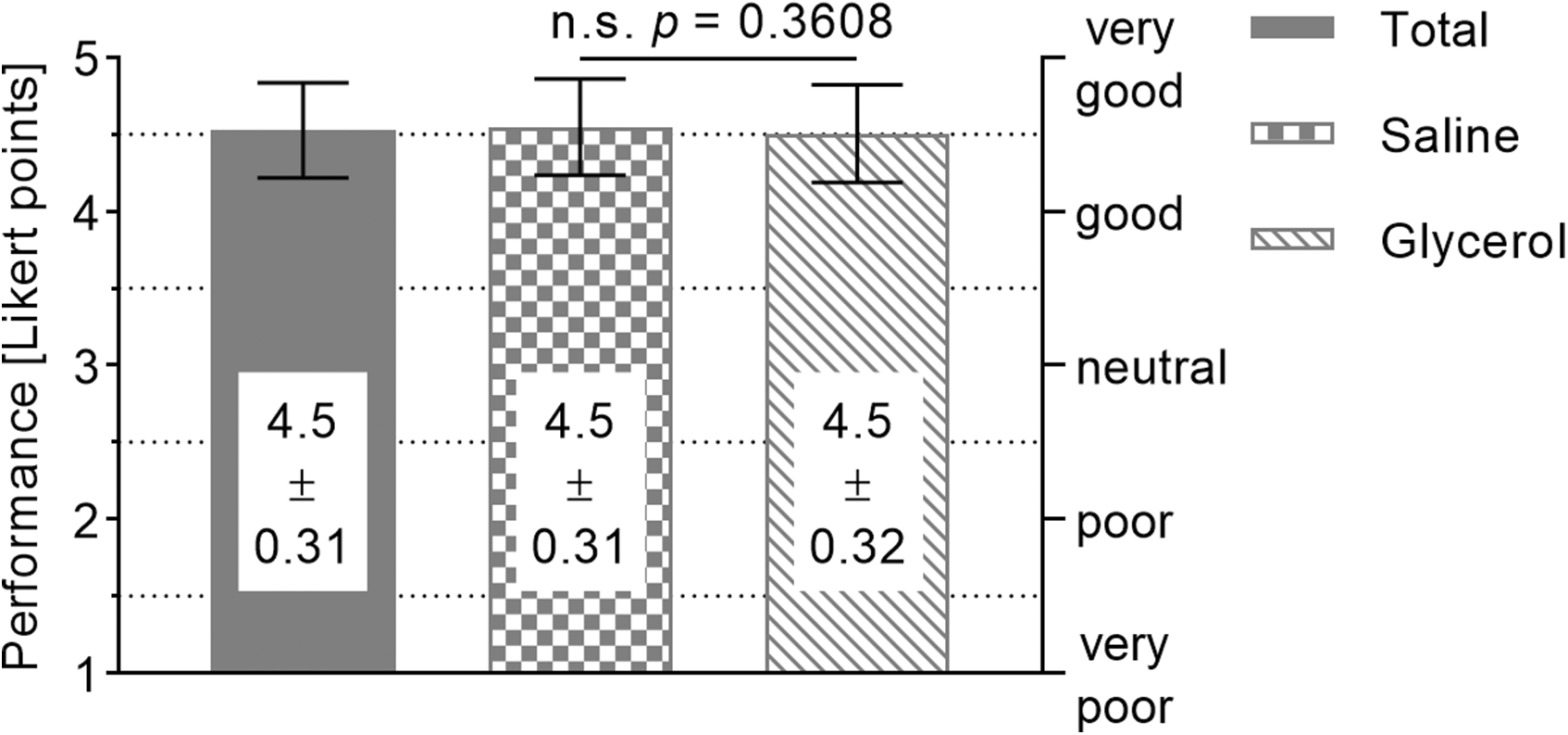
Mean overall performance scores [± standard deviation] using the HYBRIDknife flex T‐Type from 28 esophageal endoscopic submucosal dissection procedures performed by four highly experienced endoscopists, 14 each with normal saline (0.9% NaCl) and fructose‐added glycerol. Performance comprised dissection properties, handling, and usability aspects.

**Figure 3:**
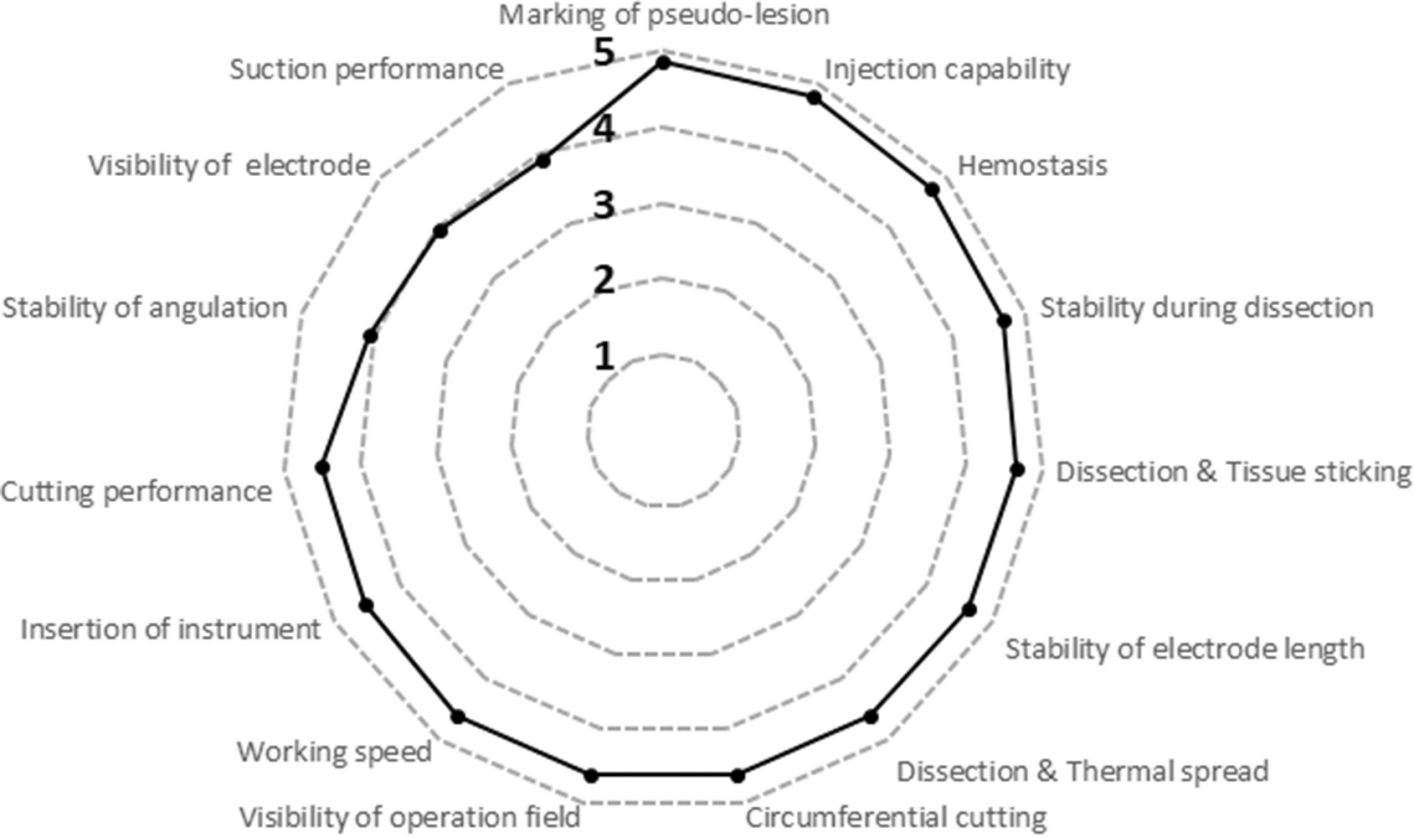
Radar chart of mean scores (*n* = 28) for each performance characteristic (Table 2) evaluated using the HYBRIDknife flex T‐Type in 28 esophageal endoscopic submucosal dissection procedures performed by four highly experienced endoscopists. Scale for each characteristic (gray dashed lines): 1 = Very poor, 2 = Poor, 3 = Neutral, 4 = Good, and 5 = Very good. Each spoke of the diagram represents one of the characteristics selected to give a comprehensive picture of the overall performance of a device for endoscopic submucosal dissection. Each data point represents the mean of 28 evaluations or endoscopic submucosal dissections.

**Figure 4:**
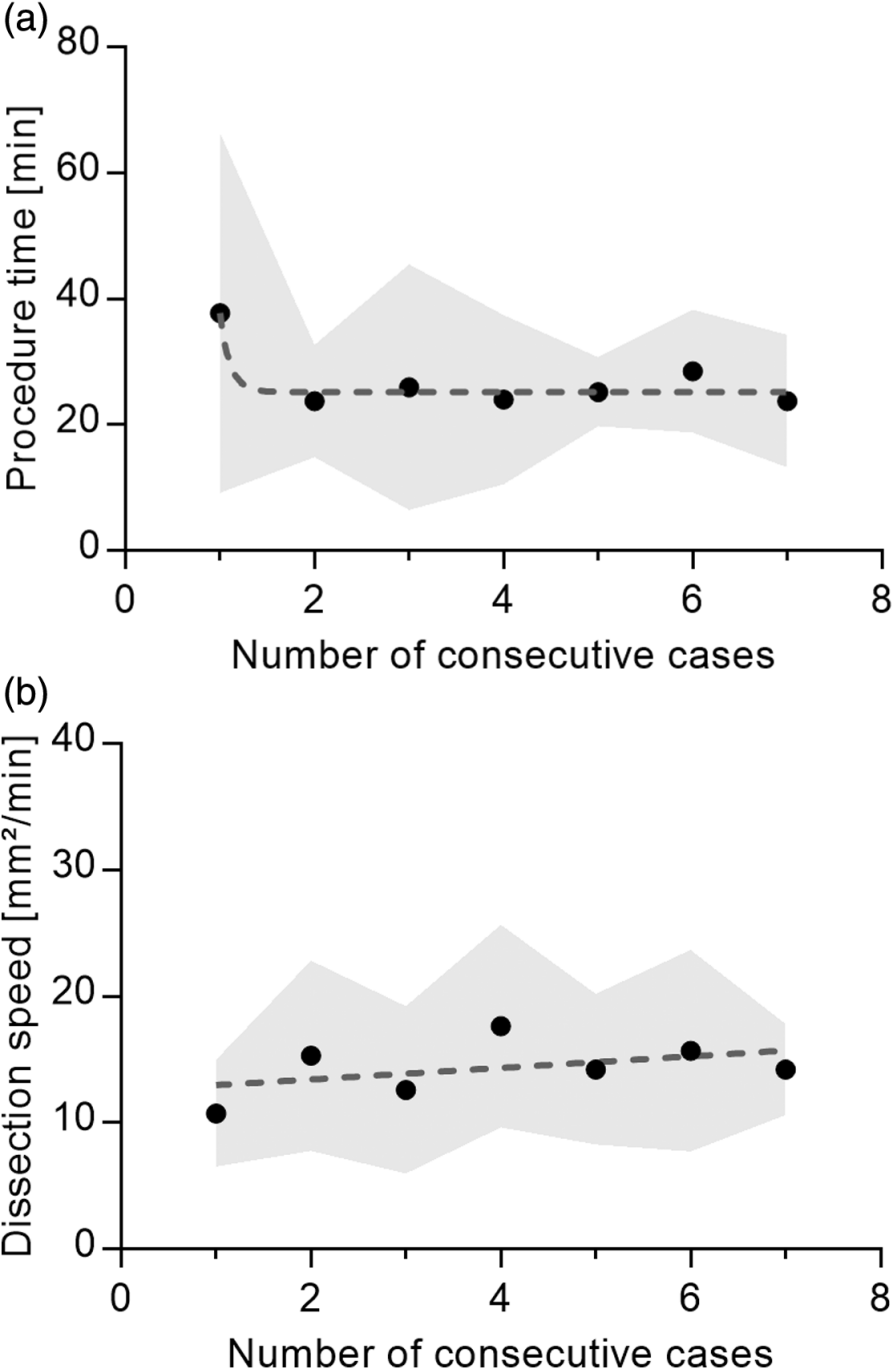
Aspects of working speed using the HYBRIDknife flex T‐Type in 28 porcine esophageal cases of endoscopic submucosal dissection, performed by four highly experienced endoscopists. (a) Decreasing mean procedure time [± standard deviation, gray shaded area] from the first compared with the remaining cases with a plateau at 25 min. (b) Nonsignificant increase in mean dissection speed [± standard deviation, gray shaded area; slope of linear regression: +0.46 mm^2^/min per consecutive case, *P* = 0.32], defined as dissection area divided by procedure time.

All 28 esophageal ESDs were completed in one piece, that is, the en bloc resection rate was 100%. No endoscopically visible perforations or major bleeding requiring intervention with hemostatic forceps occurred. Minor bleeding that could be controlled by coagulation with HK‐T occurred in 0 of 14 (0%) cases in the normal saline group and in 1 of 14 (7.1%) cases (with more than five bleeding episodes) in the glycerol group, which was not a significant difference (*P* > 0.99). Thus, there were no instrument changes for hemostasis. The HK‐T instrument itself was replaced once in 1 of 28 (3.6%) ESDs due to a technical failure (the tip was damaged). The HK‐T was used for needle‐free high‐pressure injection in each ESD case (28 of 28, 100%). Mean overall performance scores were similar for saline and glycerol (Fig. 2; saline: 4.5 ± 0.31 LP vs. glycerol: 4.5 ± 0.32 LP, *P* = 0.36). The mean performance score of all 28 ESD cases was 4.5 ± 0.31 LP (Fig. 2). Individual comparisons of the 15 aspects of performance (Table 2) revealed no significant advantages of one injection fluid over the other (range of mean differences: 0–0.3, range of median differences: 0–0.5, range of *P*‐values: 0.105–1). To illustrate the results regarding the aspects of performance, the mean scores of all 28 ESD cases are shown (Fig. 3). These scores ranged between the optimum of 5 LP and 4.5 LP in terms of pseudo‐lesion marking (4.9 ± 0.36 LP), injection capability (4.8 ± 0.39 LP), hemostasis (4.7 ± 0.35 LP), stability during dissection (4.7 ± 0.46 LP), tissue sticking during dissection (4.7 ± 0.48 LP), stability of electrode length (4.6 ± 0.49 LP), thermal spread during dissection (4.6 ± 0.57 LP), circumferential cutting (4.6 ± 0.50 LP), visibility of the working field (4.6 ± 0.50 LP), working speed (4.6 ± 0.50 LP), ease of instrument insertion (4.5 ± 0.51 LP), and overall cutting performance (4.5 ± 0.51 LP). Lower, but still acceptable, were the scores for stability of angulation with inserted instrument (4.1 ± 0.77 LP), electrode visibility (4.0 ± 0.74 LP), and suction performance with inserted instrument (3.9 ± 0.79 LP). Regarding suction performance, it should be noted that the gastroscope had a working channel of 2.8 mm, the outer diameter of the HK‐T tube was 2.1 mm, and the distal tip of the HK‐T had a diameter of 2.6 mm. The procedure duration was similar in both groups (saline: 26 ± 13 min vs. glycerol: 28 ± 16 min, *P* = 0.45). Overall, the mean procedure time was 27 ± 15 min. In addition, we observed a decrease in mean procedure time from the first cases of each endoscopist to the subsequent cases (Fig. 4a). The dissection speed was similar in the saline and glycerol groups (saline: 13 ± 6.2 mm^2^/min vs. glycerol: 15 ± 6.1 mm^2^/min, *P* = 0.22). Altogether, the mean dissection speed was 14 ± 6.1 mm^2^/min. Regarding a learning curve, we detected a slight but nonsignificant increase in dissection speed across consecutive cases (*P* = 0.32 of linear regression, Fig. 4b).

## Discussion

In this prospective *in vivo* porcine study, esophageal ESDs were performed with the new HK‐T, with 14 ESDs, each using either saline or glycerol for submucosal injection. Performance was rated on a five‐point scale, with 1 being the worst and 5 the best. No endoscopic perforations or major bleeding occurred. All resections were completed en bloc. For both injectates, the mean device performance and dissection speed were high at 4.5 LP and 14 mm^2^/min, respectively. The outstanding features of the HK‐T included precise electrosurgical cutting and coagulation with a thin 0.5‐mm electrode and reliable high‐pressure injection with minimal risk of bleeding, which does not require a conventional injection needle.

ESD will become increasingly important for the effective treatment of esophageal cancer.^24^ In our study, no procedure‐related perforations or major bleeding occurred, most likely due to the physicians' extensive experience in ESD. Publications of porcine esophageal ESD cases suggest a 0%–26% risk of major bleeding and a 0%–11% perforation risk.^9^, ^11^ The porcine cases do not directly reflect clinical risks. For example, in humans, the perforation rate was approximately 4% until 2003,^25^ but a range of 0%–5.2% was reported subsequently as techniques and experience in ESD became more established.^8^, ^26^, ^27^ Differences in health status and experimental vs. clinical setting should also be considered. Nonetheless, the pig represents one of the most useful models of the human GI tract, providing endoscopic and surgical access close to the clinical setting.^28^ Among other models of digestive disease, porcine esophageal metaplasia and neoplasia are being studied because they resemble the clinical and pathological aspects of human disease,^29^ suggesting that the porcine model does not underestimate complication rates.

We evaluated various performance aspects related to HK‐T utilization. Porcine and human cases with the established HybridKnife suggest that dissection performance and speed are better with glycerol compared with saline injection.^13^ We observed no improvements with glycerol, but two major differences between that study and ours should be considered: the endoscopists' experience with ESD (relatively little experience, 15 human and 50 pig cases vs. 10–20 years of experience) and the anatomical site (gastric antrum/body vs. esophagus). We surmised that the difference in device performance and procedure speed cannot be explained by the device, as the new HK‐T is technically superior compared with the established HybridKnife. It would therefore appear that ESD experience is the main predictor of working speed. One limitation that may have affected working speed and performance was the transition to the HK‐T, as there was no opportunity for training in the use of this new device before the study commenced. The average procedure time was longer for the first ESD cases than for the other cases (38 min vs. 25 min, Fig. 4a). There was also a slight, but nonsignificant, increase in dissection speed between the first and subsequent cases (11 mm^2^/min vs. 15 mm^2^/min, Fig. 4b). We interpret the initial decrease in procedure time as a general but rapid adaptation to the less familiar setting in the porcine cases, while the slight increase in dissection speed could represent a learning effect despite the endoscopists' experience in ESD. Overall, there appeared to be a smooth transition from the more familiar ESD systems used clinically by the endoscopists, to the new HK‐T system.

In addition to physiological saline, various fluids have been proposed for submucosal injection, such as sodium hyaluronate,^30^ fructose‐added glycerol,^30^ hypertonic saline‐epinephrine,^30^ glucose solution with added epinephrine,^31^ or 50% dextrose solution.^30^ The main selection factor for such a fluid is its higher viscosity compared with saline, which promises longer‐lasting mucosal elevation. Other important factors are availability and cost. In our study, glycerol was chosen because it offers an advantage regarding long‐lasting submucosal lifting^30^, ^32^ and is widely used in East Asia.^32^, ^33^, ^34^ We performed esophageal ESDs safely with excellent procedural success using the HK‐T either with saline or glycerol. Despite the theoretical advantages of submucosal injection with glycerol,^13^, ^30^ our study showed no significant improvements in the safety and performance metrics examined. It is possible, though, that the relatively small resection size did not permit a significant difference to be shown. Endoscopists who are practically at the end of their learning curve can use experience to overcome various difficulties that may arise during an ESD. Trainees may benefit more as they are at the beginning of their learning curve, but this needs confirmation by clinical studies. Combining HK‐T hybrid technology with a viscous injection fluid could be an additional technical improvement to make difficult ESDs easier to perform. The main limitations of our pilot study were the small number of cases and the restriction to animals. Another limitation was the nonsurvival study design, where delayed perforation due to deep thermal injury of the muscularis propria may have been missed.

In conclusion, we have reported here our initial experience with esophageal ESD performed in a live porcine model using a novel flexible electrosurgical knife that allows unique high‐pressure through‐needle submucosal injection. We were able to show that esophageal ESD can be performed safely and rapidly with this device, and with excellent performance evaluation by the endoscopists. Both saline and glycerol may be used for high‐pressure submucosal injection with the HK‐T system, with equal safety and efficacy.

## Ethics statement

Animal Studies: The study was approved by the Institutional Animal Care and Use Committee (IACUC), Singapore (IACUC Ref No.: 2022/SHS/1748, 19‐Oct‐2022) and conducted in accordance with the “Guide for the Care and Use of Laboratory animals”, published by the National Academy of Sciences.
